# When Less or More Isn't Enough: Renal Maldevelopment Arising From Disequilibrium in the Renin-Angiotensin System

**DOI:** 10.3389/fped.2019.00296

**Published:** 2019-07-17

**Authors:** Lucas Ferreira de Almeida, Terezila Machado Coimbra

**Affiliations:** Department of Physiology, Ribeirão Preto Medical School, University of São Paulo, Ribeirão Preto, Brazil

**Keywords:** angiotensin II, fetal programming, kidney development, renin-angiotensin system, vitamin D deficiency

## Abstract

Environmental and nutritional factors during fetal and neonatal life can have long-lasting effects on renal functions and physiology and susceptibility to kidney disease in adulthood. All components of the renin-angiotensin system (RAS) are highly expressed in the kidneys during the period of renal development. The RAS plays a central role in the regulation of various cellular growth factors and stimulates adhesion molecules and cellular migration. The use of antagonists of this system during fetal development represents a major risk factor for hypertension, renal vascular dysfunction, and kidney medulla atrophy in adulthood. The inappropriate activation of the RAS by vitamin D (VitD) deficiency has been studied in recent years. Clinical and experimental studies have demonstrated an inverse relationship between circulating VitD levels and blood pressure, plasma and renin activity, and an increase in angiotensin II and the receptor AT_1_. These data raise new questions about the importance of the integrity of the RAS during development since RAS pathway inhibitors and VitD deficiency have opposing functions. This is a literature review on the possible mechanisms by which antagonists of the RAS and VitD deficiency during fetal development provoke disturbances in kidney structure and function. Potential mechanisms are presented and discussed, and the possible pathways by which an imbalanced maternal RAS may negatively impact fetal development and have consequences in adulthood are also explored.

## Kidney Development: Humans and Rodents

Several histologic studies (1–6) provide the basis for our contemporary knowledge about human renal development. These works demonstrated the appearance of key anatomical features and provide a comparative histological view derived from cross-species studies of the kidneys in humans, cats, chicks, mice, and other mammals (1–6). These anatomical studies emphasized the conserved evolutionarily features of mammalian renal developmental as well as the differences between species. Some studies performed in rodents (mice and rats) have provided a mechanistic framework for mammalian renal development (7–9).

In humans, renal development is initiated at approximately 4 weeks of pregnancy and is completed late in gestation. Most (60–65%) nephrons are formed during the third trimester of pregnancy (10). After this period, no new nephrons are formed throughout the lifetime of the individual. Despite that Rodríguez et al. (11), have reported that the kidney continues to form after extreme preterm birth, being the glomerulogenesis altered in this case, characterized by fewer glomeruli in the kidneys. In rodents (rats and mice), important structures of the kidney develop during the lactation period (P1–P14) (12). Renal development proceeds in the subcapsular region until postnatal day 7 (P7) to 8 and is continued through the growth and functional maturation of the kidney medulla (12). The loop of Henle proliferates by mitotic activity, in the kidney medulla, peaking around P14, and reaching functional and structural maturation in the fourth week (13).

Recently, genetic, molecular and cellular studies have provided detailed insights into the cell types and molecular and cellular processes involved in kidney development (14, 15). They demonstrated various conserved features between mice and humans, including overall structures, nascent nephron arrangement, and distinct cell lineage markers. Since rodents are born with immature kidneys, neonatal rat, and mouse models are used as animal's models to study the mechanisms of renal development in humans (15).

## Renin-Angiotensin System (RAS) Inhibition Impairs Renal Development in Rodents

**Figure 1:**
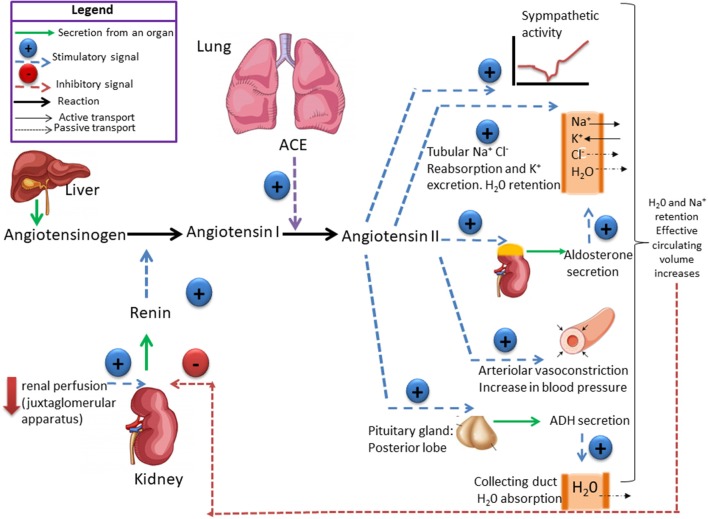
The renin-angiotensin system (RAS) is responsible for the maintenance of salt and water balance. Renin catalyses the conversion of angiotensinogen into angiotensin I which is converted by the angiotensin-converting enzyme (ACE) into angiotensin II (ANGII). ANG II controls the secretion of aldosterone, which stimulates Na^+^ retention and antidiuretic hormones (ADH) that stimulates H_2_O reabsorption by the kidney. Plasma volume and plasma osmolality controls salt appetite and drinking behavior. High-Na^+^ intake leads to change in both plasma volume and osmolality, which exert a negative feedback on renin secretion. Ultimately, high-Na^+^ consumption induces a decrease in aldosterone concentration, which reduces Na^+^ reabsorption and increases sodium excretion. High-Na^+^ intake does not induce change in ADH plasma concentration, if the intake of H_2_O is enough to maintain a Na^+^ and H_2_O balance.

The RAS plays a fundamental role in controlling tissue perfusion, arterial blood pressure, and extracellular volume (16). Activation of this system starts by the synthesis and secretion of angiotensinogen, the precursor polypeptide, in the liver (17). This circulating preprohormone is cleaved by renin, an enzyme synthesized in, and released from the juxtaglomerular cells in the kidney afferent arterioles, into angiotensin I (ANGI) (18). The inactive intermediate decapeptide ANGI is further cleaved by the angiotensin converting enzyme (ACE), a peptidase located on the luminal surface of endothelial cells and lung, into the octapeptide angiotensin II (ANGII) (18). ANGII acts through two receptor subtypes, the AT_1_ and the AT_2_ receptors (Figure 1). ANGII contributes to vascular homeostasis by increasing vascular tone acting directly on ANGII receptors and indirectly by enhancing sympathetic adrenergic function to increase vascular tone (19). Acutely, this is necessary to maintain adequate perfusion pressure in patients with hypovolemia or reduced cardiac output (19). At longer term (e.g., hours to days), ANGII contributes to vascular homeostasis by its effect on extracellular fluid volume (20). It stimulates the adrenal cortex to release aldosterone, that acts on the kidneys to increase Na^+^ and fluid retention (20). ANGII also stimulates the release of antidiuretic hormone (ADH) from the posterior pituitary, causing the kidneys to increase fluid retention (21) (Figure 1). Disruption of this system may cause increase in the arterial pressure, with damage of target organs, including the kidneys.

Studies over the last two decades reveal the crucial importance of RAS in early kidney development (22–26). Dysfunctions caused by the blockage of the RAS during renal development period are characterized by glomerular volume reduction (22, 23), tubular dilatation (22), and papillary atrophy (12) with an increase in the relative interstitial area of the renal cortex (22), a reduction in urinary concentration capacity (25), and a decrease in the urinary glomerular filtration rate (22). Machado et al. (23) observed that animals exposed to the blockage of the RAS during renal development exhibited glomerulosclerosis and severe albuminuria during adulthood (10 months of age). We demonstrated that AT_1_ receptor blockade during lactation causes glomerular disorders that are characterized by the presence of small (immature) glomeruli in adulthood, associated with albuminuria and hypertension (22). Madsen et al. (12) reported deterioration of the renal microvasculature and vasa recta bundles in animals that received RAS blockers for up to 2 weeks after birth. They suggest that ANGII can promote the postnatal growth of peritubular capillaries through the AT_1_ receptor and that renal postnatal development is linked to angiogenesis regulation. Yoo et al. (27) used RAS blockers during pregnancy to analyze the pathways that lead to disturbances in the renal microvasculature. They observed an imbalance between pro- and antiangiogenic factors associated with renal vascular rarefaction.

Epithelial-to-mesenchymal transition (EMT) and its reverse process, mesenchymal-to-epithelial transition (MET), have been implicated in several diseases and in development (28). In renal disorders, an imbalance in homeostatic crosstalk between tubular epithelial cells and interstitial mesenchymal cells (or fibroblasts) leads to a complete reorganization of the tubulointerstitial region (29). RAS inhibition during renal development was shown to repress the differentiation of proximal tubule epithelial cells by inducing EMT. There is increased markers for mesenchymal cells, such as α-smooth muscle actin (α-SMA) and vimentin, and a decrease in the cubilin receptor present in the brush border, a marker for cell differentiation (22). A recent study demonstrated that lymphangiogenesis is impaired in rats exposed to a RAS blocker on postnatal day 8 (30). The authors suggest that tubulointerstitial region damage may be due to a disrupted RAS, at least in part, because of impairment of the MET/EMT signaling pathways. Taken altogether, (a) the disrupted interaction between mesenchymal and tubular cells, (b) the increased fluid and excess of macromolecules in the interstitium impairing lymphatic drainage, (c) and the rarefaction of peritubular capillaries, may increase the risk of progressive renal injury in these experimental models.

## RAS Blockade During Pregnancy Leads to Disturbances in Human Fetal Development

Blockers of the RAS are efficient and widely accepted as antihypertensive drugs (31, 32). However, the use of RAS blockers during the second and third trimesters of pregnancy should be avoided. *In utero*, fetuses are susceptible to exposure to RAS blockers, as they are associated with fetopathy characterized by intrauterine growth retardation, amniotic fluid reduction, renal dysplasia, oliguria, renal injury, and death (26, 33, 34). Nadeem et al. (35) reported an association between intrauterine exposure to RAS blockers and acute kidney injury and chronic kidney disease in a retrospective study of 24 children. The researchers observed severe renal disturbances in children exposed to RAS blockers during the second and/or third trimesters compared with children exposed during the first trimester of pregnancy. These observations corroborate previous findings from clinical, laboratory, and epidemiologic studies (36, 37). However, infants exposed to angiotensin receptor antagonists (ARBs) during the first trimester presented an increased risk of major congenital malformation compared to infants who had no exposure to these medications (38). A poor outcome was reported for ABRs use compared to ACE inhibitors (37), suggesting that the development of fetal disturbances in urine production is a gradual process that occurs after the first trimester of pregnancy (36). In addition, some congenital malformations, such as skull-ossification defects and patent ductus arteriosus, have been reported with the use of RAS blockers and have been attributed to the subsequent effects of fetal renal disorder (34, 39, 40). Hypertension in women and preeclampsia has increased substantially worldwide. According to the NHANES survey from 2011 to 2014, 85.7 million people over the age of 20 in the USA, more than half of which are women, have hypertension (41). Thus, the treatment of hypertension by RAS blockers has increased considerably. Although the US Food and Drug Administration (FDA) issued a black-box warning in 1992 cautioning against the use of RAS blockers during the second and third trimesters of pregnancy, the percentage of pregnant women exposed to RAS blockers was almost three times greater in 2003 than in 1986–1988 (42).

In addition to kidney disorders caused by exposure to medications that interfere with RAS during renal development such as ACE inhibitors or AT_1_ receptor blockers, genetic mutations of the RAS genes in humans leads to renal tubular dysgenesis (RTD), a severe disturbance described by prematurity, anhydramnios, severe hypotension, and neonatanal renal failure (43–45). Gribouval et al. (46) studied 11 individuals with RTD, from nine families, and found that they had homozygous or compound heterozygous mutations in the genes encoding renin, angiotensinogen, ACE or AT_1_ receptor. RTD is a heterozygous inherited autosomal-recessive disease with over 50 registered mutations (47–50).

## Expression and Functions of RAS Components During Kidney Development

A cascade of events during kidney development is tightly regulated by ANGII receptors, which are highly expressed in the kidneys during this period (51). Thus, the integrity of the RAS plays a key role in fetal health. Kidney development is a highly complex process demanding a precise regulation of cellular proliferation, differentiation, and apoptosis (36, 52).

**Figure 2:**
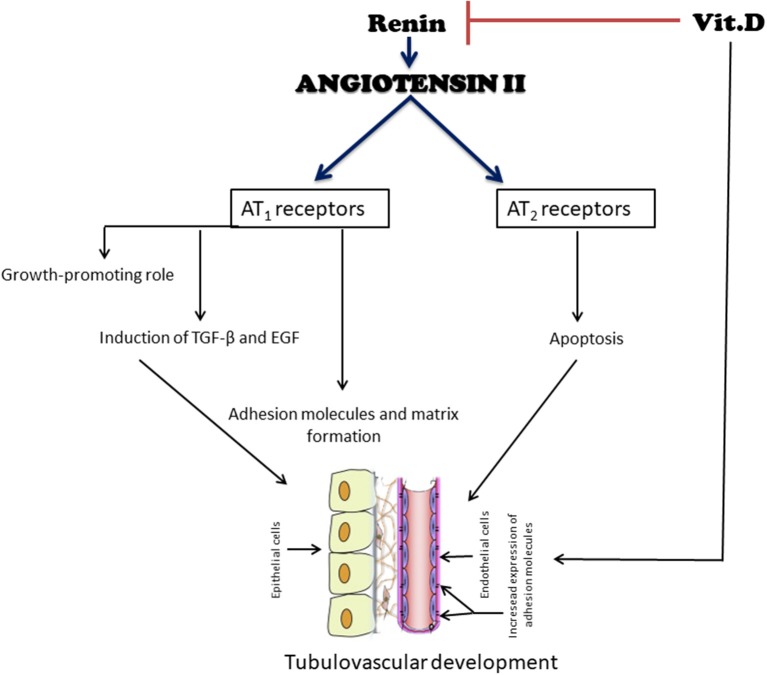
Simplified diagram of the effects of angiotensin II (ANGII) and vitamin D (VitD) on tubulovascular development. ANG II exerts a growth-stimulatory (hypertrophy, proliferation, and migration) effect through the activation of AT1 receptors. In contrast, the binding of ANG II to AT2 receptors may induce apoptosis, an important event during renal development, to remove excess extracellular matrix and undifferentiated cells. Another function of ANG II is the induction of growth factors, such as transforming growth factor β (TGF-β) and epidermal growth factor (EGF). ANG II also stimulates components of the extracellular matrix and adhesion molecules. Their coordinated expression is a prerequisite for a successful tubular vascular development. VitD negatively modulates the RAS by suppressing the renin gene. It also has both direct and indirect roles in the cell cycle and cell proliferation, differentiation, and apoptosis, all important for an adequate renal development.

The temporal and spatial distribution of AT_1_ and AT_2_-type ANGII receptors and their mRNAs during renal development has been previously studied (53, 54). An increase in the mRNA for AT_1_ receptor can be detected in renal glomeruli, vessels, and cortex in newborn rats, when cell proliferation and differentiation occur simultaneously (51). Kakuchi et al. (54) reported the presence of AT_2_ mRNA in mice in the interstitium, which is in contact with the collecting ducts and, to a lesser extent, with the nephrogenic mesenchyme, providing evidence of its participation in the mesenchymal-epithelial differentiation and apoptosis, important events during renal development (Figure 2). The relationship between apoptosis and the presence of these receptors results in the removal of mesenchyme excess (53). Song et al. (55) hypothesized that abnormal collecting system development in AT_2_ receptor-deficient mice is, at least partially, due to the dysregulation of ureteric bud branching morphogenesis as well as aberrant ureteric bud cell proliferation and apoptosis. The absence of AT_1_-receptor stimulation interferes with the proliferation/apoptosis balance through different pathways. In support of this notion, our group demonstrated that neonatal losartan treatment in rats leads to an increased apoptosis (25, 56) and cell proliferation, evaluated by proliferating cell nuclear antigen (PCNA), in adulthood (22). In addition to directly stimulate cell growth, ANGII regulates the synthesis of several important growth factors for normal renal development, as well as the expression of transforming growth factor (TGF-β) and epidermal growth factor (EGF) through the AT_1_ and AT_2_ pathways (57) (Figure 2). A recent work with genome-wide assessment identified pathways that are differentially regulated by ANGII, including those that have key roles in ion transport, metabolism, immune responses, apoptosis, and cell proliferation (58).

Svitok et al. (59) directly analyzed the effects of increased ANGII during the prenatal period in offspring. ANGII was administrated to pregnant rats via osmotic mini-pumps and analyzed postnatal development via blood pressure control. The animals exhibited hypertension and decreased salt sensitivity compared to controls. Prenatal treatment leads to increased aldosterone levels and decreased plasma renin activity, suggesting a complex physiological response to ANGII. These effects lead to the RAS upregulation during development, compromising the fetus cardiovascular system and exerting a long-term influence on offspring health.

## Vitamin D Deficiency During Pregnancy Adversely Affects Renal Development

The importance of RAS in early kidney development was already discussed but there is recent evidence that vitamin D (VitD) also plays a physiological role in renal development and its disturbances (22). Since the fetal source for VitD comes exclusively from maternal stores, VitD levels should remain optimal during pregnancy. The major form of VitD in humans is vitamin D3 or cholecalciferol, which is synthetized in the skin after exposure to sunlight or ultraviolet light (60). It can also be obtained from nutritional sources, especially fatty fish. It is hydroxylated in the liver into 25-hydroxyvitamin D_3_ (25(OH)D) and again hydroxylated in the kidney by 1-alpha-hydroxylase [1α(OH0ase] into 1,25-dihydroxyvitamin D3 (1,25(OH)_2_D_3_) that is the biologically active form VitD (61). This active form of VitD stimulates the calcium absorption from the intestine (62). Once that 1,25(OH)_2_D_3_ is adequately available, 24,25-dihydroxyvitamin D (24,25(OH)_2_D) is formed in the kidney, which is further catabolized (62).The VitD metabolites are bound in the circulation to the VitD-binding protein (VDBP) which has a strong affinity to 25(OH)D, 24,25(OH)_2_D and 1,25(OH)2D. The VDBP is also structurally very similar to albumin (63).The active metabolite 1,25(OH)_2_D_3_ enters the cell and binds to the VitD-receptor (VDR). This complex forms a heterodimer with the retinoid receptor and binds to a VitD responsive element on a responsive (VDREs) gene (63).

Lately the functions of VitD beyond calcium metabolism have been demonstrated (64, 65). More than 200 genes with VDREs directly or indirectly influence cell cycling and proliferation, differentiation, apoptosis and regulation of the renin gene, which are important aspects of development (66–69). VitD can enhance the expression of several brush border proteins. It induces the expression of occludin and claudin, which are junctional adhesion molecules (JAMs), and connexins (gap junctions), which basically function as cell-adhesion molecules (70, 71). It also mediates cell-to-cell and/or cell-to-extracellular matrix adhesive interactions (72, 73). These are all important events for renal development (Figure 2).

The occurrence of VitD deficiency in Western societies has reemerged as a public health issue (74). A systematic review reported the increased prevalence of low levels of VitD in Southern Europe and Eastern Mediterranean regions, despite the presence of abundant sunshine (75). A recent study demonstrated that VitD deficiency in pregnant women has become a common issue (76).

Boyce et al. (77) observed that, upon VitD deficiency in maternal rats, fetal renin expression was upregulated, and is sustained throughout adult life. Nascimento et al. (78) analyzed the first two generations of mouse offspring exposed to maternal VitD deficiency during renal development. They demonstrated that such pups exhibited hypertension and increased renal expression of renin and AT_1_ receptor. They also showed that these disturbances were transmitted to the second filial F2 generation. The question of how developmental programming passes to subsequent generations deserves further attention. Severe maternal protein deficiency leads to chronic disease in adulthood, affecting both the F1 and F2 generations (79) and extending to the F3 generation (80).

Both Maka et al. (81) and Nascimento et al. (78) reported glomeruli maturation disturbances in mouse and rat models of VitD deficiency during development and suggested that these glomeruli may be functionally impaired. In addition, we recently demonstrated vascular rarefaction, impaired nitric oxide (NO) production and hypertension in female pups from VitD-deficient mothers as consequences of renal dysfunction (82). We suggested that these alterations are partially due to the imbalanced RAS caused by VitD deficiency. These data corroborate findings from the literature demonstrating that VitD downregulates renin expression (68, 83) via the inhibition of cAMP response element-binding protein (CREB), which is required for the expression of renin (84). VDR knockout mice and mice deficient in VitD develop high blood pressure, hyperreninemia, cardiac hypertrophy, and other renal dysfunctions (68).

The vascular endothelium from VitD-deficient offspring has an impaired ability to relax due to a disturbance in the production of two important vasodilator factors: NO and endothelium-derived hyperpolarizing factor (85). Wei et al. (86) showed that the deletion of the VDR in endothelial cells leads to endothelial dysfunction with disturbances in blood vessel relaxation and that these mice are more responsive to the hypertensive effect of ANGII infusion. These disturbances in blood pressure provoked by VitD deficiency, associated with endothelial vasodilator impairment, cannot be explained by low serum calcium because these studies did not find a difference between the deficient groups and controls (82, 85).

Several clinical studies have demonstrated a correlation between plasma VitD levels and endothelial function, as assessed by flow-mediated vasodilation (87), and the amelioration of endothelial function with VitD supplementation in patients (88, 89). The improvement of endothelial function may be due to the increased expression/activity of NO synthase 3 (eNOS) and the higher production of NO along with the inhibition of increased oxidative stress caused by ANGII (90, 91). One of the possible pathways through which VitD leads to increase NO production is the phosphorylation of the intracellular p38/MAPK, P13/Akt, and ERK1/2 pathways, leading to eNOS activation (91). Thus, it is clear that maintaining physiological VitD levels during pregnancy and adulthood is fundamental for an adequate vascular development and function.

Mice lacking the VDR develop pronounced polyuria, or excessive urinary output, suggesting that VitD plays an important role in fluid homeostasis (68). VDR null mutant mice present polyuria, with increased renin and ANGII in the kidney and brain compared with wild-type mice, leading to marked increase in water intake and salt appetite (92). The polyuria observed in VDR null mutant is not related with disturbances in renal fluid handling or increased intestinal salt absorption but rather is the consequence of increased water intake induced by the increase in systemic and brain ANGII. A recent study demonstrated that 1α(OH)ase null mutant mice also presented polyuria, suggesting that VitD may regulate production and excretion of urinary in kidney (93). The authors reported that 1α(OH)ase null mutant mice presented hypertension, increased renin, ANGII and AT_1_ receptor levels and increased malondialdehyde in the brain, together with compromised antioxidant expression. These disturbances were improved with 1,25(OH)_2_D_3_ administration, demonstrating that VitD is able to regulate central RAS activation via a central antioxidant mechanism.

## Conclusion

In this review, we pointed out and discussed the role of the RAS, the importance of its integrity during renal development and the relationship between the RAS and VitD, emphasizing on its non-calcemic functions. Components of the RAS pathway have a vital role in early kidney development. The inhibition of this system using RAS antagonists causes several abnormalities in renal development, some of which are related to VitD disturbances. A tight regulation of the RAS pathway is needed to support normal kidney development and function. RAS pathway inhibitors and VitD deficiency have opposing functions and VitD deficiency leads to overactivation of RAS pathways, inducing kidney alterations during development that are permanent in adult life. Finally, non-calcemic effects of VitD include direct and indirect interference on vascular cells through the RAS modulation and several studies pointed out that serum level of this vitamin is important for the good health of pregnant women and their children.

## Author Contributions

TC: responsible for critically revising the manuscript and given final approval of the version to be published. LdA: responsible for conception and design, literature review, drafting the manuscript and for given final approval of the version to be published.

### Conflict of Interest Statement

The authors declare that the research was conducted in the absence of any commercial or financial relationships that could be construed as a potential conflict of interest.
